# Modeling temporal genetic variability using mixed models improves yield stability and selection efficiency in *Coffea canephora*

**DOI:** 10.3389/fpls.2026.1840043

**Published:** 2026-05-12

**Authors:** Alex Campanharo, Deurimar Herênio Gonçalves Júnior, Máskio Daros, Leandro Mendel da Cruz, Isabel Marques, Raphael Ricon de Oliveira, Fábio Luiz Partelli

**Affiliations:** 1 Federal University of Espírito Santo (UFES), North University Center of Espírito Santo (CEUNES), São Mateus, Espírito Santo, Brazil; 2 Minas Gerais State Company for Technical Assistance and Rural Extension (EMATER-MG), Pocrane, Minas Gerais, Brazil; 3 Capixaba Institute for Research, Technical Assistance and Rural Extension (INCAPER), Local Rural Development Office of Muniz Freire, Parque de Exposições, Centro, Muniz Freire, Espírito Santo, Brazil; 4 Forest Research Centre (CEF), Associate Laboratory TERRA, School of Agriculture, University of Lisbon, Lisbon, Portugal; 5 Department of Biological Sciences, State University of Santa Cruz (UESC), Ilhéus, Bahia, Brazil

**Keywords:** climate variability, genetic correlations, genetic covariance structures, genotype-by-year interaction, longitudinal data, perennial crop breeding, REML/BLUP, temporal yield stability

## Abstract

**Introduction:**

Increasing climatic variability challenges Coffea canephora breeding programs to identify genotypes that combine high productivity with temporal stability across contrasting seasons.

**Methods:**

We evaluated 44 genotypes across four consecutive crop seasons (2022–2025) in eastern Minas Gerais, Brazil, using mixed linear models (REML/BLUP) with seven alternative variance-covariance structures for genetic and residual effects.

**Results:**

The flexible model M7 (unstructured genetic covariance matrix with year-specific residual variances) provided the best fit (lowest AIC and BIC). Plot-level heritability ranged from 0.64 to 0.68, genotype-mean heritability from 0.84 to 0.86, repeatability was 0.89, and selective accuracy ranged from 0.92 to 0.94. Genetic correlations revealed atypical behavior in 2023, driven by heat stress during grain filling. Relative selection efficiency increased cumulatively by 4.4% after four years. Genotypes Bicudo, A1, and AD1 combined the highest predicted genotypic values with elevated persistence indices.

**Discussion:**

Flexible mixed model approaches improve the reliability of genetic evaluation and support resilience-oriented selection strategies in C. canephora, enabling accelerated genetic gain and identification of superior genotypes adapted to variable cultivation conditions.

## 1 Introduction

Increasing climatic variability has intensified yield instability in coffee production systems, challenging the capacity of breeding programs to identify genotypes capable of sustaining high and stable productivity across contrasting years (Tavares et al., 2018; Ferreira et al., 2024). Across several producing regions, climate-driven heat and water stress have been associated with recurrent yield declines and marked interannual variability, with losses often reaching double-digit percentages (Pham et al., 2019). For instance, prolonged drought combined with recurrent temperature extremes in Brazil has reduced productive potential in key coffee-growing regions, leading to downward adjustments in 2025/26 output projections and contributing to price volatility in international markets^1^. In perennial crops, where selection relies on repeated multi-year evaluations, such instability intensifies genotype × year interaction and increases the likelihood of recommending genotypes whose performance is contingent on transient environmental conditions (Fernandes Filho et al., 2025).

*Coffea canephora*, commonly known as Robusta or Conilon coffee, accounts for more than 40% of global coffee bean production^2^. Its relevance stems from its tolerance to high temperatures, adaptation to low-altitude environments, and relative resistance to pests and diseases (Ferrão et al., 2024). Brazil is the second largest producer of the species and holds a substantial genetic heritage in germplasm collections, while continuously investing in breeding programs, thereby establishing itself as a global reference in the development of new cultivars (Jordaim et al., 2025; Adunola et al., 2024; Ferrão et al., 2020). Beyond direct physiological effects on plant metabolism and fruit development, climate change is also expected to alter the distribution and severity of pests and diseases, including the coffee leaf miner (*Leucoptera coffeella*) and fungal pathogens such as Hemileia vastatrix, further compounding yield instability and reinforcing the need for resilience-oriented selection strategies (Harelimana et al., 2022).

Brazilian breeding programs aim to sustainably increase productivity and production efficiency (Baqueta et al., 2024). However, in perennial crops such as *C. canephora*, increasing interannual climatic variability complicates the identification of genotypes that combine high productivity with resilience across contrasting seasons. Therefore, genotypes selected based on performance in a limited number of crop years may fail to sustain yield under subsequent climatic conditions, increasing the risk of productivity losses at the field level. Conversely, reducing the number of crop seasons to lower costs and accelerate selection cycles may compromise the accuracy of genetic estimates due to strong environmental effects (Adunola et al., 2023; Fernandes Filho et al., 2025). In this context, the estimation of genetic parameters such as repeatability and heritability is essential to balance cost, accuracy, and selection gain (Roka et al., 2024).

Traditionally, analysis of variance (i.e. ANOVA) and mixed linear models under REML/BLUP in their simplified forms have been employed to define the number of harvest years and to assess genotype-by-year interaction in coffee breeding (Mistro et al., 2008; Rocha et al., 2015). Nonetheless, these approaches assume homogeneous variances and restricted correlations across years, assumptions that are often unrealistic under variable climatic conditions. Such simplifications may obscure biologically meaningful differences in genotype performance and limit the capacity to correctly characterize temporal patterns of genetic stability. Longitudinal analyses of yield stability in *C. canephora* clones have confirmed that covariance structures such as CS, CSH, and UN provide adequate fit to multi-year data in this species (Cilas et al., 2011), and that temporal instability is a consistent feature of production even across extended evaluation periods of up to 14 years (Cilas et al., 2003). More recently, Covre et al. (2022) applied a Bayesian MCMC framework to 43 C*. canephora* genotypes evaluated across four harvests in two contrasting environments and reported broad-sense heritability of 0.28, highlighting the challenges of precise genetic estimation when temporal covariance structure is not explicitly modeled. In contrast, the use of mixed models incorporating alternative variance-covariance structures allows greater flexibility in modeling heterogeneous variances and non-uniform correlations enabling greater precision in the genetic evaluation of perennial crops such as coffee (Henderson, 1975; Patterson and Thompson, 1971; Piepho et al., 2008; Evangelista et al., 2023).

Despite these methodological advances, systematic comparisons among different variance-covariance structures in *C. canephora* remain scarce. Most analyses rely on simplified models, which restrict the understanding of the complex genotype-by-year interactions in this perennial species (Bergo et al., 2020). The adoption of more flexible structures, capable of accommodating heterogeneous variances and non-uniform correlations represents critical, yet still underexplored, step toward improving selection decisions in *C. canephora* breeding programs.

Therefore, understanding genotype-by-year interaction is crucial for identifying superior genotypes and determining the appropriate number of crop seasons required for reliable and resilient selection. Accordingly, the objectives of this study were to: (i) compare variance-covariance structures in *C. canephora*; (ii) estimate key genetic parameters relevant to yield stability and resilience; (iii) determine the minimum number of years required for reliable selection and resilient selection; and (iv) identify genotypes combining high productivity with persistence under variable climatic conditions in order to enhance breeding strategies for *C. canephora* in Brazil.

## 2 Materials and methods

### 2.1 Experimental area and cultivation conditions

The experimental area was located in the municipality of Aimorés, in the eastern region of Minas Gerais, Brazil, at 19°34′54.79″ S latitude, 41°23′00.99″ W longitude, and approximately 260 m altitude. The predominant soil type was classified as dystrophic Red-Yellow Latosol, according to the Brazilian Soil Classification System corresponding to the Oxisol in USDA Soil Taxonomy and Ferralsol in WRB (Santos et al., 2025).

**Figure 1:**
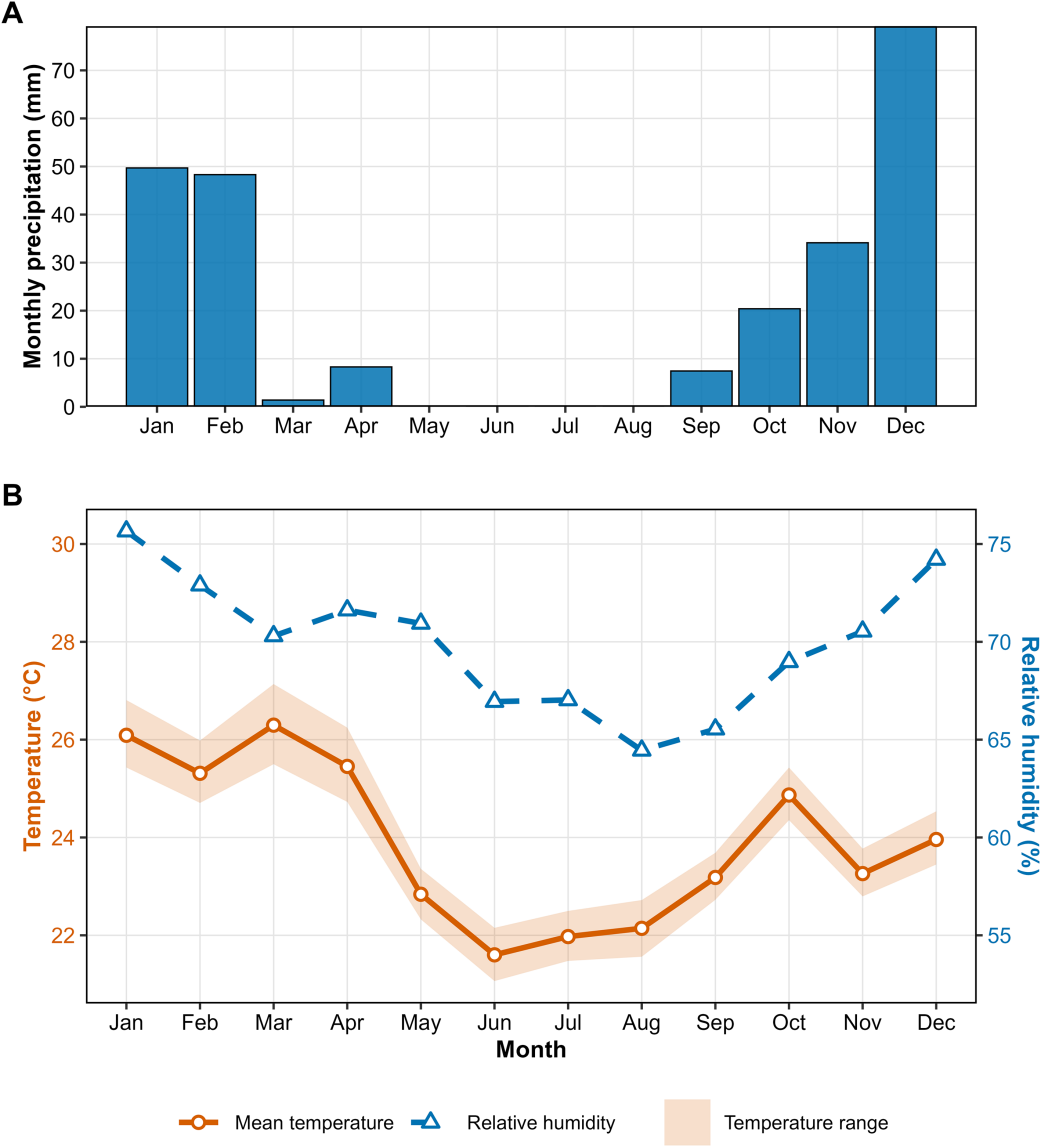
Monthly climatic conditions during the experimental period (2022–2025). **(A)** Total monthly rainfall. **(B)** Mean air temperature (solid line), monthly temperature range (shaded area), and mean relative humidity (dashed line). Data was recorded at the experimental site.

The regional climate is tropical, characterized by hot and humid summers and dry winters, and classified as Aw under the Köppen system (Alvares et al., 2013; Beck et al., 2023). During the experimental period (2022-2025), the mean air temperature was 24.1 ± 3.9 °C (mean ± standard deviation), with absolute maximum and minimum temperatures of 36.1 °C and 13.0 °C, respectively. Total precipitation reached 862 mm, with an annual average of 216 mm. The mean relative humidity was 70.4 ± 16.8% (**Figure 1**)^3^. Notably, the 2023 growing season was characterized by recurrent high-temperature episodes during grain filling, providing a natural contrast for evaluating genotype performance under heat stress.

### 2.2 Genetic material and experimental design

A total of 44 *Coffea canephora* genotypes were evaluated, cultivated at a spacing of 3.2 m between rows and 0.8 m between plants (approximately 3,906 plants ha^-1^), with two orthotropic stems per plant. The genotype set comprised three groups (**Table 1**): two Embrapa cultivars (category a), 23 promising Conilon genotypes originating from traditional producing regions in Espírito Santo (category c), and 19 seed derived selections from eastern Minas Gerais (category b named LMG). These LMG genotypes (category b) were initially identified as seed derived selections in farmers’ fields and were subsequently clonally propagated for inclusion in the trial. All genotypes were established simultaneously and evaluated at the same plant age. Detailed identification and classification of the evaluated genotypes is presented in **Table 1**.

**Table 1:** Identification and classification of the 44 *Coffea canephora* genotypes evaluated in Aimorés, Minas Gerais, Brazil.

Identification	Cultivar reference	Identification	Cultivar reference
BRS 125^a^	Embrapa	CH1 ^c^	Farmer selection from Espírito Santo
BRS 88 ^a^	Embrapa	Imbugudinho ^c^	Monte Pascoal (Partelli et al., 2024)
LMG1^b^	Selection in eastern Minas Gerais	AD1 ^c^	Plena (Partelli et al., 2024)
LMG2 ^b^	Selection in eastern Minas Gerais	Graudão HP ^c^	Salutar (Partelli et al., 2024)
LMG3 ^b^	Selection in eastern Minas Gerais	Valcir P ^c^	Farmer selection from Espírito Santo
LMG4 ^b^	Selection in eastern Minas Gerais	Beira Rio 8 ^c^	Tributum (Partelli et al., 2024)
LMG5 ^b^	Selection in eastern Minas Gerais	AP ^c^	Monte Pascoal (Partelli et al., 2024)
LMG6 ^b^	Selection in eastern Minas Gerais	L80 ^c^	Plena (Partelli et al., 2024)
LMG7 ^b^	Selection in eastern Minas Gerais	Bamburral ^c^	Tributum (Partelli et al., 2024)
LMG8 ^b^	Selection in eastern Minas Gerais	Pirata ^c^	Tributum (Partelli et al., 2024)
LMG9 ^b^	Selection in eastern Minas Gerais	Peneirão ^c^	Monte Pascoal and Plena (Partelli et al., 2024)
LMG10 ^b^	Selection in eastern Minas Gerais	Ouro Negro ^c^	Farmer selection from Espírito Santo
LMG11 ^b^	Selection in eastern Minas Gerais	A1 ^c^	Andina, Tributum, and Plena (Partelli et al., 2024)
LMG12 ^b^	Selection in eastern Minas Gerais	P2 ^c^	Monte Pascoal (Partelli et al., 2024)
LMG13 ^b^	Selection in eastern Minas Gerais	P1 ^c^	Andina (Partelli et al., 2024)
LMG14 ^b^	Selection in eastern Minas Gerais	LB1 ^c^	Monte Pascoal and Plena (Partelli et al., 2024)
LMG15 ^b^	Selection in eastern Minas Gerais	Clementino ^c^	Tributum (Partelli et al., 2024)
LMG16 ^b^	Selection in eastern Minas Gerais	Verdim TA ^c^	Andina (Partelli et al., 2024)
LMG17 ^b^	Selection in eastern Minas Gerais	K61 ^c^	Farmer selection from Espírito Santo
LMG18 ^b^	Selection in eastern Minas Gerais	Guarani ^c^	Forte Guarani (Partelli et al., 2024)
LMG19 ^b^	Selection in eastern Minas Gerais	MP3 ^c^	Farmer selection
Bicudo ^c^	Plena (Partelli et al., 2024)	JN ^c^	Farmer selection

^a^ Embrapa cultivars. ^b^Seed derived selections from eastern Minas Gerais (LMG genotypes). ^c^Promising Conilon genotypes originating from traditional producing regions in Espírito Santo.

The experiment was conducted using a randomized complete block design, with 44 treatments (genotypes) and three replications (blocks or experimental plots). Each plot consisted of five plants, with only the three central plants evaluated to minimize border effects. Crop management followed technical recommendations for coffee cultivation. Harvests were carried out annually from 2022 to 2025, totaling four consecutive crop seasons.

Productivity was determined based on the harvest of ripe fruits from each experimental plot, conducted separately by genotype. The harvested fruit volume was initially measured in liters per plot and subsequently converted to 60-kg bags of processed coffee per hectare. For this conversion, an equivalence of 320 liters of ripe fruits per 60-kg bag of processed beans was considered, with adjustments made according to plant density per hectare based on the adopted spacing.

### 2.3 Statistical analyses

Analyses were performed using the methodology of mixed linear models. The basic model considered was:


$$y=1\mu +X_{1}a+X_{2}b+Z_{1}g+Z_{2}p+e$$

where y represents the n×1 vector of phenotypic observations; μ is the overall mean associated with the vector of ones; a is the vector of fixed effects of years with incidence matrix $X_{1}$; and b is the vector of fixed effects of replications within years, associated with incidence matrix $X_{2}$. The term g corresponds to the vector of random genotypic effects, assumed as $g∼N(0,{\text{Σ}}_{g}⊗I_{44})$, where ${\text{Σ}}_{g}$ is the 4×4 matrix of genetic variances and covariances across years, and $I_{44}$ is $Z_{1}$. The term p is the vector of random permanent plot effects ( q×1), assumed as $p∼N(0,\sigma_{p}^{2}I_{q})$, where $\sigma_{p}^{2}$ is the variance of permanent environmental effects and $I_{q}$ is the identity matrix of order 132 (number of plots), associated with incidence matrix $Z_{2}$. Finally, e is the vector of random errors, assumed as $e∼N(0,{\text{Σ}}_{e}⊗I_{nj})$, where ${\text{Σ}}_{e}$ is the matrix of residual variances across years and $I_{nj}$ is the identity matrix of order $n_{j}$ (number of observations per year).

### 2.4 Modeling of genetic effects

The matrix ${\text{Σ}}_{g}$ was modeled with four alternative covariance structures, while two approaches were considered for the residual matrix ${\text{Σ}}_{e}$, yielding seven models in total. These structures were defined *a priori* as a systematic progression from the most restrictive to the most flexible parameterisation applicable to repeated-measures data in plant breeding (Gilmour et al., 2009; Butler et al., 2023), covering the principal options available for modeling ${\text{Σ}}_{g}$ and ${\text{Σ}}_{e}$. This progression was not derived from preliminary data screening but reflects the theoretical framework for longitudinal mixed models, in which progressively relaxing constraints on variances and correlations allows an empirical assessment of which assumptions are supported by the data.

Initially, a baseline model was fitted, adopting a homogeneous compound symmetry structure, which partitions the genetic variance into the main effect and the effect due to genotype-by-year interaction (GYI):


$$\Sigma_{g}=[\begin{array}{cccc} \sigma_{g}^{2}+\sigma_{gy}^{2} & \sigma_{g}^{2} & \ldots & \sigma_{g}^{2}\\ \sigma_{g}^{2} & \sigma_{g}^{2}+\sigma_{gy}^{2} & \ldots & \sigma_{g}^{2}\\ \vdots & \vdots & \ddots & \vdots \\ \sigma_{g}^{2} & \sigma_{g}^{2} & \cdots & \sigma_{g}^{2}+\sigma_{gy}^{2} \end{array}]⊗I_{t}$$

where t is the number of genotypes ( t=44). This structure treats all crop seasons as exchangeable environments, assuming that the genetic expression of yield is equally variable and equally correlated across any pair of years, an assumption equivalent to a traditional repeatability model.

The second covariance structure was the diagonal (DIAG), which accounts for heterogeneity of genetic variances across years while assuming null paired covariances:


$$\Sigma_{g}=[\begin{array}{cccc} \sigma_{g1}^{2} & 0 & 0 & 0\\ 0 & \sigma_{g2}^{2} & 0 & 0\\ 0 & 0 & \sigma_{g3}^{2} & 0\\ 0 & 0 & 0 & \sigma_{g4}^{2} \end{array}]⊗I_{t}$$

By allowing year-specific genetic variances while fixing all covariances at zero, DIAG acknowledges that the magnitude of genetic expression can differ across seasons but assumes that the relative ranking of genotypes is entirely independent between any two years, a biologically extreme assumption for a perennial crop evaluated under variable but related climatic conditions.

The next covariance structure was heterogeneous compound symmetry (CSH), which allows heterogeneous genetic variances across years while assuming a common correlation ( ρ) between any pair of years:


$$\Sigma_{g}=[\begin{array}{cccc} \sigma_{g1}^{2} & \rho \sigma_{g1}^{2}\sigma_{g2}^{2} & \rho \sigma_{g1}^{2}\sigma_{g3}^{2} & \rho \sigma_{g1}^{2}\sigma_{g4}^{2}\\ \rho \sigma_{g2}^{2}\sigma_{g1}^{2} & \sigma_{g2}^{2} & \rho \sigma_{g2}^{2}\sigma_{g3}^{2} & \rho \sigma_{g2}^{2}\sigma_{g4}^{2}\\ \rho \sigma_{g3}^{2}\sigma_{g1}^{2} & \rho \sigma_{g3}^{2}\sigma_{g2}^{2} & \sigma_{g3}^{2} & \rho \sigma_{g3}^{2}\sigma_{g4}^{2}\\ \rho \sigma_{g3}^{2}\sigma_{g1}^{2} & \rho \sigma_{g4}^{2}\sigma_{g2}^{2} & \rho \sigma_{g4}^{2}\sigma_{g3}^{2} & \sigma_{g4}^{2} \end{array}]⊗I_{t}$$

CSH relaxes the variance constraint of CS while retaining a single correlation parameter shared across all year pairs, reflecting the assumption that the degree of genetic consistency between seasons is constant regardless of which two years are being compared.

Finally, to more flexibly account for heterogeneity of genetic variances, an unstructured (UN) model was fitted. This structure imposes no constraints on either variances or pairwise correlations, allowing the data to determine freely how genotypic expression varies across seasons and how consistently genotypes perform between any specific pair of years. It is the biologically most realistic option for perennial crops where each crop season integrates a unique combination of climatic conditions, bearing cycle stage, and cumulative plant developmental effects.


$$\Sigma_{g}=[\begin{array}{cccc} \sigma_{g1}^{2} & \sigma_{g12}^{2} & \sigma_{g13}^{2} & \sigma_{g14}^{2}\\ \sigma_{g21}^{2} & \sigma_{g2}^{2} & \sigma_{g23}^{2} & \sigma_{g24}^{2}\\ \sigma_{g31}^{2} & \sigma_{g32}^{2} & \sigma_{g3}^{2} & \sigma_{g34}^{2}\\ \sigma_{g41}^{2} & \sigma_{g42}^{2} & \sigma_{g43}^{2} & \sigma_{g4}^{2} \end{array}]⊗I_{t}$$

### 2.5 Modeling of residual effects

Two main approaches were considered for modeling residual effects. In the first case, homogeneity of residual variances across years was assumed, using the identity structure:


$$R=\sigma_{e}^{2}I_{n}$$

where $\sigma_{e}^{2}$ is the common residual variance and $I_{n}$ is the identity matrix of order n.

In a second step, models with heterogeneous residuals were fitted, allowing specific variances for each year. In this case, the residual matrix assumes a heterogeneous diagonal form:


$$R=[\begin{array}{cccc} \sigma_{e1}^{2} & 0 & 0 & 0\\ 0 & \sigma_{e2}^{2} & 0 & 0\\ 0 & 0 & \sigma_{e3}^{2} & 0\\ 0 & 0 & 0 & \sigma_{e4}^{2} \end{array}]$$

where $\sigma_{ej}^{2}$ represents the residual variance estimated for the *j*-th year.

### 2.6 Model selection

Model fit was evaluated using the Akaike Information Criterion (AIC; Akaike, 1974) and the Bayesian Information Criterion (BIC; Schwarz, 1978). The AIC is defined as


A IC=−2log L+2t

where L denotes the maximum value of the (restricted) likelihood function and t is the number of estimated parameters. The BIC was computed as


BIC=−2log L+tlog(n)

with n representing the number of observations. In both cases, smaller values indicate a more parsimonious balance between model fit and complexity.

### 2.7 Estimation of genetic and non-genetic parameters

The estimation of variance components and genetic parameters was performed using mixed models, considering both the baseline model (M1) and the best-fitting model. From the variance components obtained, several genetic parameters of interest were calculated.

The individual phenotypic variance was defined as the sum of genetic variance, genotype-by-year interaction variance, permanent environmental variance, and residual variance:


$$\sigma_{ph}^{2}=\sigma_{g}^{2}+\sigma_{gy}^{2}+\sigma_{p}^{2}+\sigma_{e}^{2}$$

The repeatability coefficient was estimated as the ratio between the sum of genetic and permanent environmental variances and the total phenotypic variance:


$$\rho =\frac{\sigma_{g}^{2}+\sigma_{p}^{2}}{\sigma_{ph}^{2}}$$

Plot-level heritability was calculated as the ratio between genetic variance and phenotypic variance:


$$h^{2}=\frac{\sigma_{g}^{2}}{\sigma_{ph}^{2}}$$

Genotype-mean heritability was obtained by considering the number of years ( a) and the number of replications ( r), according to the following expression:


$$h_{mg}^{2}=\frac{\sigma_{g}^{2}}{\sigma_{g}^{2}+\frac{\sigma_{gy}^{2}}{a}+\frac{\sigma_{p}^{2}}{r}+\frac{\sigma_{e}^{2}}{ar}}$$

Cullis heritability (Cullis et al., 2006) was estimated from the average variance of prediction errors ( $\bar{V}({\text{Δ}}_{g})$) of pairwise genotype differences:


$$H_{Cullis}^{2}=1-\frac{\bar{V}(\Delta_{g})}{2{\hat{\sigma }}_{g}^{2}}$$

Selective accuracy was obtained following Mrode (2014), calculated as:


$$r=\sqrt{1-\frac{PEV}{\sigma_{g}^{2}}}$$

where PEV represents the prediction error variance associated with each genotype. Parameters were estimated both in the baseline model and in the best-fitting model for comparative purposes. When the best-fitting model presented a heterogeneous structure, values of $H^{2}$ and r were defined separately for each year.

Genetic correlations across years were derived from the variance-covariance matrices of genotypic effects fitted under different structures. In the case of the unstructured (UN) model, the genetic correlation between years jand $j^{\prime }$was estimated as the ratio between the genetic covariance and the product of the square roots of the marginal genetic variances:


$$\rho_{g}=\frac{\sigma_{jj^{\prime }}}{\sqrt{\sigma_{j}^{2}\;\sigma_{j^{\prime }}^{2}}},$$

where $\sigma_{jj^{\prime }}$ represents the genetic covariance between years jand $j^{\prime }$, and $\sigma_{j}^{2}$ and $\sigma_{j^{\prime }}^{2}$ are the specific genetic variances for each year.

Relative selection efficiency was estimated based on the cumulative repeatability of yield across years, following the approach proposed by Resende (2002). For this purpose, the genotypic variance-covariance matrix across years was constructed, from which the average repeatability was obtained. Efficiency was then calculated as:


$$Efficiency_{i}=\sqrt{\frac{i}{1+(i-1)\rho }}$$

where i represents the number of years considered and ρthe cumulative repeatability.

Yield persistence of genotypes was estimated from the predicted genotypic values ( ${\hat{g}}_{ij}$) obtained from the best-fitting model (Rocha et al., 2018). For each year ( j), the ideotype was defined as the highest BLUP observed ( $\text{max}({\hat{g}}_{j})$). Subsequently, for each genotype ( i), the sum of squared differences between its predicted value and the ideotype across years was calculated. The persistence measure was obtained as the ratio between this sum and the total accumulated across all genotypes, expressed on a relative scale:


$$P_{i}=\frac{\sum_{j=1}^{m}{\left({\hat{g}}_{ij}-max({\hat{g}}_{j})\right)}^{2}}{\sum_{i=1}^{v}\sum_{j=1}^{m}{\left({\hat{g}}_{ij}-max({\hat{g}}_{j})\right)}^{2}}$$

where m corresponds to the number of years evaluated and v to the number of genotypes. Higher values of $P_{i}$ indicate greater proximity of the genotype to the ideotype across years, reflecting higher yield stability and persistence.

All analyses were implemented in the RStudio integrated development environment (IDE), using the R programming language (version 4.5.1; R Core Team, 2025). The ASReml-R package (Butler et al., 2023), version 4.2.0.355, was employed for model fitting, while ggplot2 (Wickham, 2016) was used for graphical visualization. A fully reproducible R script detailing data processing, mixed model specification, model updating, and the computation of all derived parameters is provided in the **Supplementary Material**.

## 3 Results

### 3.1 Model selection

Model comparison revealed clear differences in goodness of fit among the seven variance–covariance structures evaluated (**Table 2**). The model combining an unstructured genetic covariance matrix with heterogeneous residual variances (M7) provided the best fit according to both AIC and BIC criteria and was therefore selected for subsequent analyses (**Table 2**). Simpler models assuming homogeneous genetic variances and correlations performed less well. Model M1, which employed compound symmetry for genetic effects and homogeneous independent residuals, yielded an AIC of 4190.61 and a BIC of 4258.92, with only three parameters estimated, serving as a reference equivalent to a traditional ANOVA model. Allowing heterogeneity of genetic variances across years (M2 and M3) did not substantially improve model fit, although M3 showed a slight improvement relative to M2.

**Table 2:** Variance-covariance structures for genetic effects ( ${\text{Σ}}_{g}$) and residuals ( ${\text{Σ}}_{e}$), values of Akaike (AIC) and Bayesian information criteria (BIC), number of parameters associated with ${\text{Σ}}_{g}$ and ${\text{Σ}}_{e}$, log-likelihood (logL), and predictive accuracy of the seven models tested in repeated-measures data analysis of *Coffea canephora*.

Model	${\text{Σ}}_{g}$	${\text{Σ}}_{e}$	AIC	BIC	${\text{Σ}}_{g}$par	${\text{Σ}}_{e}$par	logL	Accuracy
M1	CS	IDV	4190.61	4258.92	2	1	-2079.31	0.92
M2	DIAG	IDV	4204.11	4280.95	4	1	-2084.06	0.92
M3	CSH	IDV	4192.77	4273.88	5	1	-2077.38	0.93
M4	UN	IDV	4176.91	4279.36	10	1	-2064.45	0.93
M5	DIAG	DIAG	4200.88	4290.53	4	4	-2079.44	0.92
M6	CSH	DIAG	4189.35	4283.27	5	4	-2072.67	0.91
**M7**	**UN**	**DIAG**	**4173.71**	**4288.98**	**10**	**4**	**-2059.86**	**0.93**

CS, homogeneous compound symmetry; DIAG, heterogeneous diagonal; CSH, heterogeneous compound symmetry; UN, unstructured; IDV, homogeneous independent residuals.Bold values indicate the best-fitting model (M7), selected based on the lowest AIC and BIC values among all models evaluated.

Models allowing greater flexibility in genetic covariance structure consistently outperformed simpler alternatives. The unstructured genetic model with homogeneous residuals (M4) substantially reduced AIC values (4176.91), while the inclusion of heterogeneous residual variances (M7) resulted in the lowest AIC among all models tested (4173.71). This difference relative to M4 indicates that modeling residual heterogeneity contributed to a more adequate fit. Among models with heterogeneous compound symmetry for ${\text{Σ}}_{g}$, M6 provided a better fit than M3, but still inferior to the unstructured models. Variance components and genetic parameters estimated under all seven models are provided in **Supplementary Table 1**.

Despite variations in information criteria, predictive accuracy was consistently high across all models, ranging from 0.91 to 0.93. This indicates that model choice primarily affected variance partitioning and biological interpretation rather than the stability of genotype rankings. Considering the balance between model fit and flexibility, M7 was selected as the most appropriate model for subsequent analyses, and all genetic parameters reported hereafter refer to estimates obtained under this model.

### 3.2 Genetic and non-genetic parameters

Estimates of variance components and genetic parameters obtained from the baseline model (M1) and the best-fitting model (M7) are presented in **Table 3**. The simplified covariance structure assumed in M1 resulted in relatively low genetic variance ( $\sigma_{g}^{2}=296.06$) and a high contribution of genotype-by-year interaction ( $\sigma_{gy}^{2}=737.33$), reflecting the limitation of this model in adequately separating genetic effects from temporal variation. In contrast, the more flexible model M7, which allowed year-specific genetic variances and heterogeneous residuals, revealed substantially higher estimates, ranging from 822.20 in 2024 to 1319.03 in 2022. Residual variances were also heterogeneous across years. This improved variance partitioning resulted in higher phenotypic variance estimates and greater consistency of genetic parameters across crop seasons.

**Table 3:** Estimates of variance components and genetic parameters obtained from the baseline model (M1) and the best-fitting model (M7) in *Coffea canephora*. Variance components are reported on the yield scale (squared units).

Component/parameter^a^	M1 2022 to 2025	M7			
		2022	2023	2024	2025
$\sigma_{g}^{2}$	296.061960	1319.0346	931.5453	822.2020	1060.9400
$\sigma_{gy}^{2}$	737.328470	–	–	–	–
$\sigma_{p}^{2}$	9.307266	10.1041097			
$\sigma_{e}^{2}$	527.839524	690.7034	433.9081	388.9035	594.1749
$\sigma_{ph}^{2}$	533.688	1559.3731	1086.2854	961.9406	1269.1024
ρ	0.56	0.89			
$h^{2}$	0.19	0.65	0.68	0.67	0.64
$h_{mg}^{2}$	0.55	0.85	0.86	0.85	0.84
$H_{Cullis}^{2}$	0.86	0.87	0.86	0.89	0.85
r	0.93	0.93	0.93	0.94	0.92
μ	83.07	114.00	78.05	64.70	75.10

**^a^**Genetic variance ( $\sigma_{g}^{2}$), genotype-by-year interaction variance ( $\sigma_{gy}^{2}$), permanent environmental variance ( $\sigma_{p}^{2}$), residual variance ( $\sigma_{e}^{2}$), phenotypic variance ( $\sigma_{ph}^{2}$), repeatability coefficient ( ρ), plot-level heritability ( $h^{2}$), genotype-mean heritability ( $h_{mg}^{2}$), Cullis heritability ( $H_{Cullis}^{2}$), selective accuracy ( r), and phenotypic mean ( μ).

Plot-level heritability ( $h^{2}$) was low under the baseline M1 (0.19), but increased markedly under M7, reaching values between 0.64 and 0.68 across years. Similarly, genotype-mean heritability ( $h_{mg}^{2}$) was also higher in the best-fitting model (0.84–0.86), compared with 0.55 in the baseline model. Cullis heritability ( $H_{Cullis}^{2}$) remained high under both models but showed greater stability across years under M7 (0.85–0.89). The repeatability coefficient ( ρ) increased from 0.56 in M1 to 0.89 ( ρ cumulative) in M7, highlighting a much higher consistency of genotype performance across years when temporal heterogeneity was explicitly modeled.

**Figure 2:**
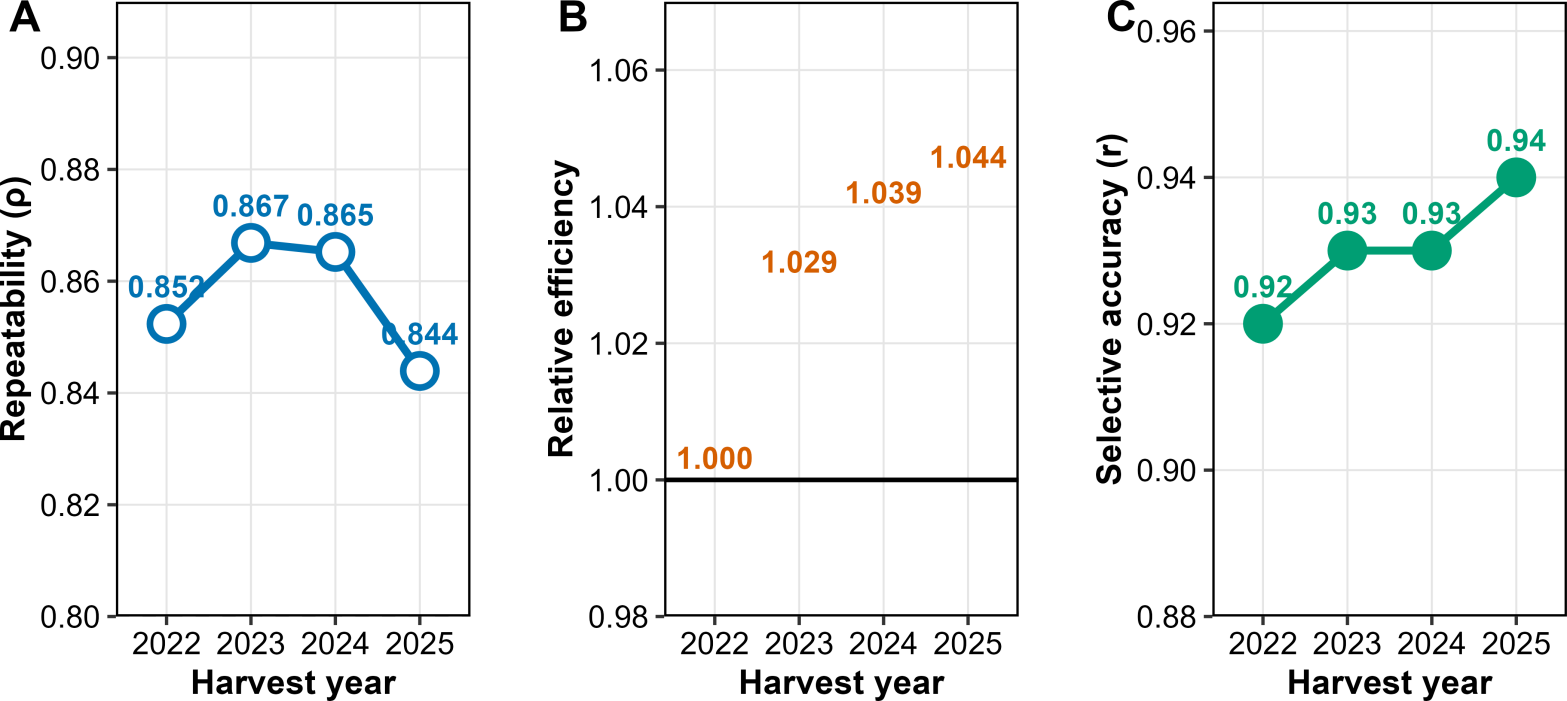
Repeatability **(A)**, relative selection efficiency **(B)**, and selective accuracy **(C)** across four harvest years for grain yield in *Coffea canephora.*.

Repeatability showed consistently high values across the four years of evaluation, ranging from 0.84 to 0.87, thereby indicating stable expression of yield across crop cycles (**Figure 2A**). Relative selection efficiency increased steadily with the addition of evaluation years, rising from 1.00 in the first year (2022) to 1.04 in the fourth year (2025), representing a cumulative gain of approximately 4.4% (**Figure 2B**). Selective accuracy remained high across all years, oscillating between 0.92 and 0.94, and confirming the reliability of the predicted genotypic values (**Figure 2C**).

**Figure 3:**
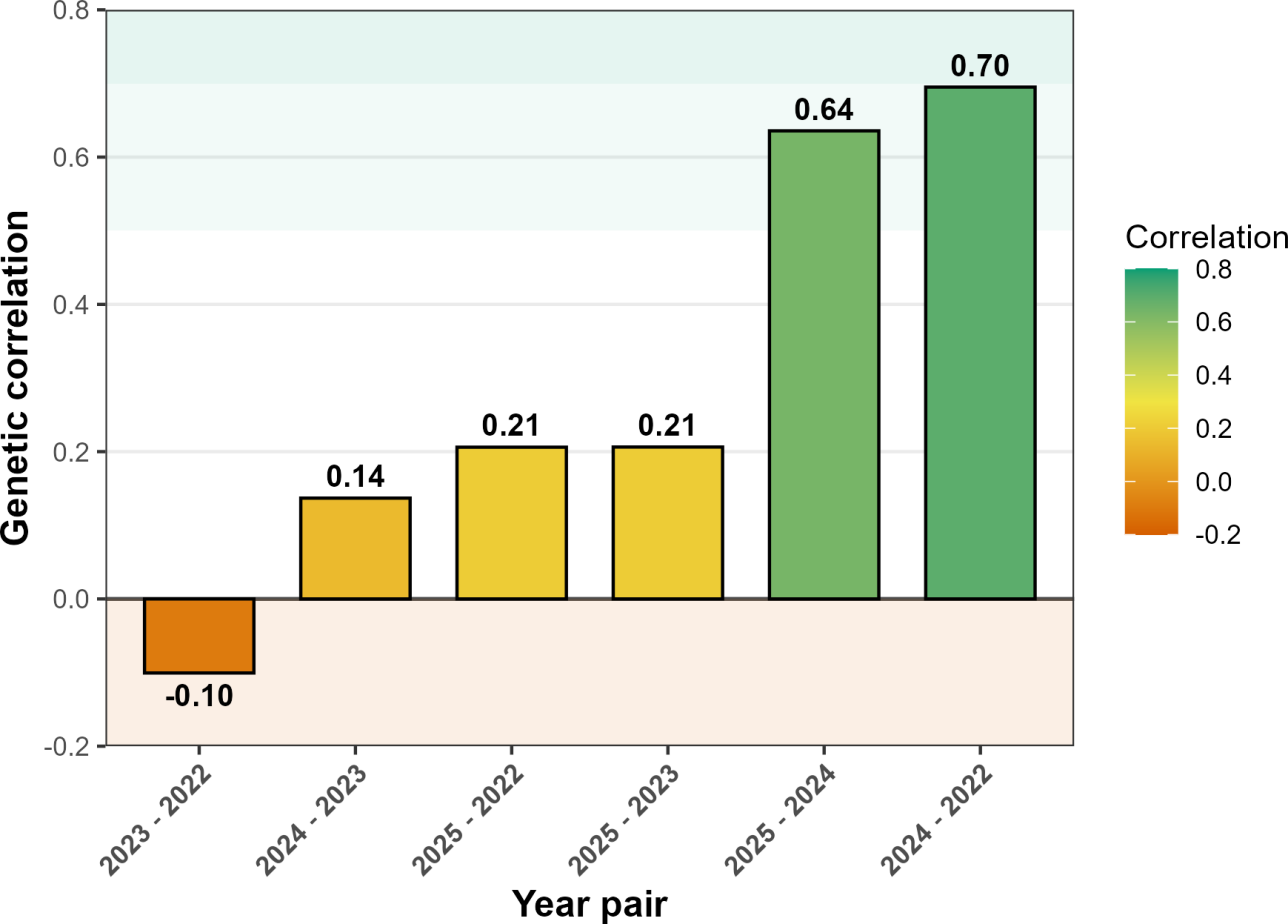
Genetic correlations for grain yield between harvest year pairs (2022–2025) in *Coffea canephora*. Bars represent correlation coefficients (r) between years.

The analysis of genetic correlations among harvest years revealed wide variation in the magnitude of coefficients (**Figure 3**). The strongest associations were observed between 2022 and 2024 (0.70) and between 2024 and 2025 (0.64), indicating greater genetic stability in these pairs of environments. In contrast, the correlation between 2022 and 2025 was low (0.21), suggesting limited consistency and reordering of genotypes. The 2023 season, characterized by extreme temperatures during grain filling, exhibited markedly reduced genetic correlations with all other years (ranging from -0.10 to 0.21), characterizing atypical behavior and strong genotype-by-environment interaction during that season.

These results indicate that 2022, 2024, and 2025 share greater genetic similarity, whereas 2023 diverges substantially from the observed pattern. From a biological standpoint, this divergence reflects differential genotypic sensitivity to the climatic conditions of that season: recurrent heat episodes above 35 °C during grain filling in 2023 likely imposed asymmetric stress across genotypes, amplifying differences in physiological buffering capacity and producing a reordering of performance ranks that no model assuming stable inter-year correlations could detect (Hasanuzzaman et al., 2013; de Oliveira et al., 2020). The detection of this anomalous season is itself a direct product of the unstructured covariance approach, under any model constraining correlations to be equal across year pairs, the atypical behavior of 2023 would have been absorbed into a pooled GxY term, obscuring rather than characterizing the interaction.

### 3.3 Selection efficiency and yield persistence

**Figure 4:**
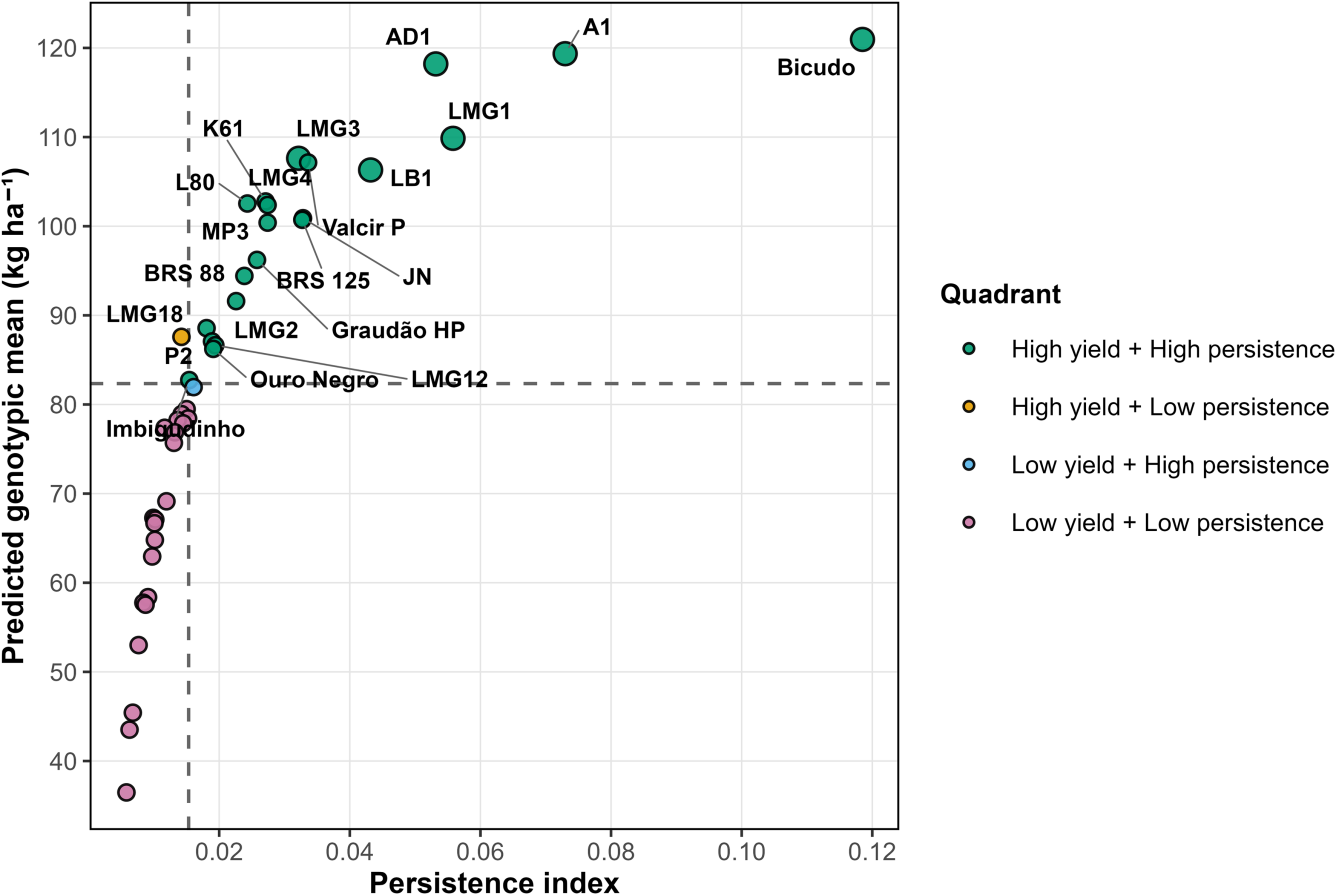
Predicted genotypic means and persistence index for grain yield *in Coffea canephora* genotypes evaluated across multiple harvest years.

The joint analysis of predicted genotypic values for yield and the persistence index enabled the identification of superior genotypes in *C. canephora* (**Figure 4**). Genotypes Bicudo, A1 and AD1, which belong to the group of promising Conilon genotypes traditionally cultivated in Espírito Santo, stood out by simultaneously exhibiting the highest predicted genotypic values (121, 119 and 118 bags ha^-1^, respectively) and elevated persistence indices, demonstrating consistent performance across years. Other genotypes, such as LMG1 and LMG3, which represent new seed-derived selections from eastern Minas Gerais, as well as Valcir P and LB1, both recognized as promising genotypes from Espírito Santo, also ranked among the most promising by combining satisfactory yield with stable performance. In contrast, genotypes LMG10, LMG14 and LMG7, all originating from the group of new seed-derived selections, showed low values for both criteria, reflecting inferior performance and reduced production regularity. These findings underscore the usefulness of jointly evaluating predicted genotypic values and persistence indices for selecting genotypes that integrate high productivity with stable performance across contrasting crop seasons.

## 4 Discussion

Repeatability studies are fundamental in the breeding of perennial species such as coffee as selection decisions rely on repeated measurements of the same genotypes over time. These long-term evaluations inevitably expose plant material to fluctuating environmental conditions, leading to heterogeneous variances and complex covariance patterns driven by genotype × environment interactions operating at multiple biological scales (Chaves et al., 2023). Under such conditions, the use of statistical models capable of accommodating this complexity becomes essential to ensure biologically meaningful interpretation and reliable genetic parameter estimation.

The comparison among variance-covariance structures underscores the need for more flexible models to capture year-to-year dynamics in *C. canephora*. The inferior performance of M2 and M3 models (**Table 2**), even with the inclusion of genetic heterogeneity, suggests that simply modeling distinct variances is insufficient to adequately represent temporal correlation. In contrast, unstructured models yielded improved statistical fit, demonstrating that genotype-by-environment interaction in perennial species rarely follows simplified patterns of homogeneity (Alves et al., 2023).

Among the models evaluated, M4 and M7 stood out, both incorporating an unstructured ${\text{Σ}}_{g}$. The additional improvement in fit observed in M7, which combined an unstructured ${\text{Σ}}_{g}$with a diagonal ${\text{Σ}}_{e}$, shows that accounting for heterogeneous residuals contributes to a more accurate description of experimental variability. Models with heterogeneous compound symmetry for ${\text{Σ}}_{g}$, such as M6, performed at an intermediate level, better than M3 but still inferior to unstructured models. Although predictive accuracy remained high across all models, the choice of M7 is justified by its superior statistical fit, greater biological consistency, and robustness for downstream inference. Importantly, this indicates that model choice primarily affects variance partitioning and biological interpretation rather than altering genotype rankings per se.

The contrast between models reveals the influence of covariance structure on the estimation of genetic parameters (**Table 3**). Model M1, by assuming homogeneity across years, substantially underestimated genetic variance, resulting in very low plot-level heritability ( $h^{2}=0.19$) and only moderate genotype-mean heritability ( $h_{mg}^{2}=0.55$). This behavior reflects a dilution of the genetic signal when temporal environmental variation is inadequately modeled. In contrast, model M7, by allowing year-specific genetic variances and heterogeneous residuals, substantially increased plot-level heritability (0.64–0.68) and genotype-mean heritability (0.84–0.86), while maintaining $H_{Cullis}^{2}$consistently high (0.85–0.89). This behavior indicates that more flexible modeling captures the data structure more realistically, reflecting the complexity of genotype-by-environment interaction.

The biological expectation of heterogeneous variances and unstructured correlations in perennial crop systems follows directly from the nature of multi-year field evaluations. Each crop season integrates a unique combination of rainfall distribution, temperature regime, bearing cycle stage, and cumulative plant developmental history, all of which affect fruit set, grain expansion, and carbon partitioning differently across genotypes. Under these conditions, assuming that genetic variance is constant across years, as in M1 and M2, or that any two seasons are equally correlated, as in CS and CSH, is biologically indefensible, not merely statistically suboptimal. The variation in genetic variance observed across years under M7 (σ²g ranging from 822.2 in 2024 to 1319.0 in 2022) reflects precisely this: in seasons where environmental conditions amplify differences in physiological buffering capacity among genotypes, the genetic signal is stronger and more reliably separated from residual noise. Longitudinal analyses in *C. canephora* have confirmed that flexible covariance structures provide better fit to multi-year yield data than restricted models (Cilas et al., 2011), and that the magnitude of genetic variance itself varies meaningfully across seasons in this species (Cilas et al., 2003).

The coherence between heritability parameters and genetic correlations reinforces this interpretation (**Figure 3**). Years 2022, 2024, and 2025 exhibited moderate to high correlations, reflecting greater consistency in genotype performance and justifying the elevated heritability estimates observed in M7. In contrast, 2023 showed very low correlations with the other years, signaling strong environmental influence and highlighting the need for models that account for heterogeneous residuals. The near-perfect repeatability in M7 ( ρ=0.89) and consistently high selective accuracy ( r=0.92–0.94), as shown in **Figure 2**, confirm that the prediction of genetic values is robust when the variance structure is properly specified.

The consistently high repeatability values observed across the four years of evaluation indicate that a large proportion of phenotypic variation is explained by permanent genetic effects, conferring greater consistency to genotype performance across crop seasons (**Figure 3**). According to Resende and Alves (2022), repeatability coefficients above 0.80 enable reducing the number of harvests required for reliable selection without compromising the accuracy of estimates. This result is corroborated by the progressive increase in relative selection efficiency, which reached a cumulative gain of 4.4% in the fourth year. Such behavior underscores the importance of multiple crop seasons to consolidate the prediction of genetic values, as also reported in studies of perennial species (Chaves et al., 2022; Dessauw et al., 2024).

Selective accuracy remained high in all years (0.92–0.94; **Figure 2C**), confirming the robustness of the estimates obtained and the reliability of the selection process. Accuracy values above 0.90 are considered highly precise and ensure that the selection reflects the true genetic merit of individuals (Cullis et al., 2006; Resende, 2016). These results demonstrate that, when the variance structure is properly specified, it is possible to shorten the evaluation period without loss of reliability, thereby increasing the efficiency of the breeding program.

Moreover, the robustness of the estimates obtained in this study derives not only from the more flexible statistical modeling (M7) but also from the quality of the balanced experimental design adopted. The use of randomized complete blocks contributed to reducing uncontrolled environmental variability, ensuring fairer comparisons among genotypes and decreasing residual variance. This experimental structure favored the attainment of more consistent heritability estimates, high repeatability, and superior selective accuracy, as observed in the results. According to Piepho et al. (2008) and Resende (2016), balanced designs maximize statistical efficiency, enhance the precision of genetic estimates, and strengthen the reliability of predictions. While longer evaluation periods may further refine parameter estimates, the present results demonstrate that robust and reliable selection can already be achieved within four crop seasons when appropriate modeling is applied.

The pattern of genetic correlations across years revealed marked heterogeneity in genotype performance, with 2023 behaving as an atypical season (**Figure 3**). The near-zero or negative correlations between 2023 and other years indicate strong genotype × environment interaction, while moderate to high correlations among 2022, 2024, and 2025 suggest greater genetic consistency during these periods. This atypical behavior is consistent with the climatic conditions recorded in 2023, when recurrent episodes of heat stress occurred, with temperatures exceeding 35 °C during the critical period of grain expansion and filling. Under such conditions, the photosynthetic apparatus may undergoes accelerated degradation, with damage to thylakoid protein complexes and reduced efficiency of photosystem II (Berry and Bjorkman, 1980; Hüve et al., 2011). Simultaneously, elevated temperatures induce stomatal closure as a protective mechanism against excessive water loss, thereby compromising CO_2_ assimilation and limiting the production of photoassimilates precisely during the phase of highest metabolic demand by the fruits (Hasanuzzaman et al., 2013). Similarly, in coffee the elevated temperatures impact metabolism, fruit production and quality (Läderach et al., 2017; de Oliveira et al., 2020; Thioune et al., 2020; Ahmed et al., 2021), which could explain the results in 2023. A Bayesian MCMC analysis of 43 C*. canephora* genotypes evaluated across four harvests in two contrasting environments reported broad-sense heritability of 0.28 (Covre et al., 2022), a value substantially lower than those obtained in the present study under M7 (0.64–0.68). This contrast suggests that the explicit modeling of temporal covariance structure within the REML/BLUP framework yields more precise partitioning of genetic variance. Covre et al. (2022) also noted that climatic variables would be strong candidates as covariates in future models, an observation that aligns with the atypical behavior of 2023 documented here and points toward a productive direction for subsequent analyses.

The joint analysis of mean predicted genotypic values and yield persistence enabled the identification of genotypes that combine high performance with greater stability across years (**Figure 4**). Genotypes such as Bicudo, A1 and AD1, all of which belong to the group of promising Conilon genotypes traditionally cultivated in Espírito Santo, consistently ranked among the most favorable, aligning with the high heritability and repeatability estimates obtained under M7. Persistence therefore captures a resilience-related property, reflecting the capacity of genotypes to maintain productive performance despite strong interannual environmental fluctuations.

This approach becomes particularly relevant when confronted with the genetic correlation patterns observed over years, especially the atypical behavior of 2023, marked by strong genotype × environment interaction. High-persistence genotypes (Bicudo, A1 and AD1) exhibited reduced sensitivity to extreme conditions, maintaining relatively stable rankings across crop cycles. The ability of a genotype to maintain its relative rank even under heat stress, as recorded in 2023, reflects physiological resilience and greater predictability of productive behavior, attributes essential for ensuring consistent economic returns to producers (Malosetti et al., 2013). This stability contrasts with materials that, although showing good performance under specific conditions, experienced drastic shifts in classification across crop cycles. In contrast, genotypes such as LMG10, LMG14 and LMG7, which represent new seed-derived selections identified in eastern Minas Gerais, in addition to presenting low predicted genotypic means, also exhibited low persistence and greater variability in correlations across years. These genotypes tend to show erratic performance, hindering reliable recommendations and increasing the risk of failure in commercial production systems.

From a practical standpoint, the selection of genotypes that integrate high-predicted yield with elevated persistence helps reduce risks associated with genotype × environment interaction and enhances breeding efficiency (Cullis et al., 2006; Resende and Alves, 2022). The integration of genetic parameters, year-to-year correlations and persistence indices provides a solid basis for safer and more sustainable selection decisions, prioritizing not only absolute productivity but also the consistency of productivity across multiple cycles and under diverse climatic scenarios. This strategy aligns with the growing need to develop resilient cultivars capable of maintaining stable yield in the face of increasingly frequent and intense climate variability.

Some limitations of this study merit explicit acknowledgement. The experiment was conducted at a single location in eastern Minas Gerais, which limits the generalizability of the genotype rankings to other production environments. The four-year evaluation window, while sufficient to demonstrate the advantages of flexible modeling and to detect the atypical behavior of 2023, does not allow definitive conclusions about long-term yield stability, longer series would be required to determine whether the patterns of genetic correlation observed here are consistent over extended periods, as evaluated for up to 14 years in diallel populations of *C. canephora* by Cilas et al. (2003). Multi-environment validation would further clarify whether genotypes such as Bicudo, A1, and AD1, identified as high-yield and high-persistence at Aimorés, maintain these attributes across the broader range of edaphoclimatic conditions encountered in Brazilian *C. canephora* production regions.

## 5 Conclusions

Flexible variance-covariance models, particularly the unstructured model with heterogeneous residuals (M7), yielded superior statistical fit and a more realistic representation of genotype-by-year interaction in *C. canephora*. By allowing year-specific genetic variances and covariances, this model produced more accurate estimates of heritability, repeatability, and selective accuracy, demonstrating that reliable selection can be conducted within shorter evaluation cycles when appropriate modeling is applied.

The atypical genetic correlations observed in 2023, driven by heat stress during grain filling, underscore the need for multiple crop seasons to reduce the risk of biased selection decisions, a conclusion that would have been inaccessible under models assuming homogeneous covariance structures. The joint analysis of predicted genotypic values and the persistence index identified Bicudo, A1, and AD1 as genotypes combining high productivity with stable performance across contrasting seasons.

These results are subject to the limitation of a single-location design and a four-year evaluation window. Multi-environment and longer-term validation would strengthen the generalizability of the genotype rankings identified here and clarify whether the climatic drivers of the 2023 anomaly operate consistently across the broader range of *C. canephora* production environments in Brazil. Incorporating climatic covariates into the modeling framework represents a natural direction for future work.

## Data Availability

The original contributions presented in the study are included in the article/**Supplementary Material**. Further inquiries can be directed to the corresponding authors.

## Footnotes

1 CONAB – Companhia Nacional de Abastecimento (2024). Brazilian National Supply Company – institutional information. Available at: https://www.abc.gov.br/training/informacoes/InstituicaoCONAB_en.aspx (Accessed March 2026).

2 International Coffee Organization (2023). *Coffee Market Report 2022–2023.* Available at: https://www.icocoffee.org/documents/cy2023-24/annual-review-2022-2023-e.pdf (Accessed March 2026).

3 Meteorological data were obtained from the Brazilian National Institute of Meteorology (INMET), historical weather database, covering the period 2022–2025.

## Supplementary material

Supplementary Material 1 Fully reproducible R script for data processing, mixed model specification, and computation of all derived parameters.
