# Supplementary material for: Modeling temporal genetic variability using mixed models improves yield stability and selection efficiency in Coffea canephora
Source: Front Plant Sci. 2026 May 12;17:1840043. doi: 10.3389/fpls.2026.1840043 (PMC13201153; doi:10.3389/fpls.2026.1840043)
Supplement: Supplementary Material 1 — Fully reproducible R script for data processing, mixed model specification, and computation of all derived parameters. [file DataSheet1.pdf]

Supplementary Material

Modeling temporal genetic variability using mixed models improves yield stability and selection efficiency in *Coffea canephora*

Supplementary Methods

Statistical Models and Variance–Covariance Structures

To account for genotype-by-year interactions and heterogeneity in genetic and residual variances across years, we fitted a series of linear mixed models using restricted maximum likelihood (REML) estimation. Models differed in their specification of genetic ($\mathbf{\Sigma}_g$) and residual ($\mathbf{\Sigma}_e$) variance–covariance structures across years.

The general form of the linear mixed model is:

$$\mathbf{y} = \mathbf{X}\boldsymbol{\beta} + \mathbf{Z}_g\mathbf{g} + \mathbf{Z}_p\mathbf{p} + \boldsymbol{\epsilon} \quad (1)$$

where $\mathbf{y}$ is the vector of phenotypic observations; $\boldsymbol{\beta}$ is the vector of fixed effects, including years and replications within years; $\mathbf{g}$ is the vector of random genotypic effects across years, assumed as $\mathbf{g} \sim \mathcal{N}(\mathbf{0}, \mathbf{\Sigma}_g \otimes \mathbf{I}_G)$, where $\mathbf{\Sigma}_g$ is the genetic variance–covariance matrix across years; $\mathbf{p}$ is the vector of random permanent plot effects; $\boldsymbol{\epsilon}$ is the vector of residual errors; and $\mathbf{X}$, $\mathbf{Z}_g$, and $\mathbf{Z}_p$ are the corresponding incidence matrices.

Seven alternative models were fitted, varying in the structure of the genetic and residual covariance matrices (Table 1).

Table 1: Alternative variance–covariance structures for genetic and residual effects across years.

**Model**	**Genetic ($\mathbf{\Sigma}_g$)**	**Residual ($\mathbf{\Sigma}_e$)**
M1	CS	IDV
M2	DIAG	IDV
M3	CSH	IDV
M4	UN	IDV
M5	DIAG	DIAG
M6	CSH	DIAG
M7	UN	DIAG

where:

- **CS**: Compound symmetry (homogeneous variances, constant correlation)
- **CSH**: Heterogeneous compound symmetry (heterogeneous variances, constant correlation)
- **DIAG**: Diagonal (heterogeneous variances, zero correlations)
- **UN**: Unstructured (heterogeneous variances, unconstrained correlations)
- **IDV**: Identity (homogeneous residual variance)

Model M1 assumes homogeneous genetic variance across years with a constant genetic correlation, and homogeneous residual variance. Model M7, the most flexible, allows for heterogeneous genetic and residual variances across years and unconstrained genetic correlations between years.

Software and Implementation

All analyses were conducted in R version 4.0 or higher (R Core Team, 2025) using the `asreml` package (Butler et al., 2023) for fitting mixed models via REML. Data manipulation was performed using the `dplyr` and `tidyr` packages. Model convergence was verified using an auxiliary function for updating ASReml models, publicly available at https://github.com/saulo-chaves/May_b_useful/blob/main/update_asreml.R.

Supplementary Code

The following R script provides a fully reproducible workflow for fitting the seven alternative mixed models and deriving genetic parameters from multi-year genotype evaluation trials.

Full R Script for Model Fitting and Parameter Estimation

```r
####################################################################
# Mixed Models for Multi-Year Genotype Evaluation Trials
# Genotype x Year Analysis with Alternative Covariance Structures
####################################################################

## 1. Clean R environment ------------------------------------------------------
rm(list = ls())
gc()

## 2. Load required packages ---------------------------------------------------
library(asreml)   # Linear mixed models (VSN International)
library(dplyr)    # Data manipulation
library(tidyr)    # Data reshaping
library(readr)    # Data import

## 3. Import data --------------------------------------------------------------
# Data file must contain the following columns:
# Genotype | Year | Rep | plot | response_variable
# where response_variable is the trait of interest (e.g., yield)

data <- read_delim("phenotype_data.txt", delim = "\t",
                   show_col_types = FALSE)

# Create unique plot identifier
data$plot <- with(data, interaction(Genotype, Rep, drop = TRUE))

## 4. Convert variables to factors ---------------------------------------------
data <- data %>%
  mutate(
    Genotype = as.factor(Genotype),
    Year     = as.factor(Year),
    Rep      = as.factor(Rep),
    plot     = as.factor(plot)
  )

## 5. Basic data checking ------------------------------------------------------
```

```r
str(data)
summary(data)
table(data$Year, data$Rep)

## 6. Source auxiliary function ------------------------------------------------
# Auxiliary function for model updating and convergence checking
# Publicly available at:
# https://github.com/saulo-chaves/May_b_useful/blob/main/fa_outs.R

source("fa_outs.R")

####################################################################
# Model Fitting ---------------------------------------------------------------
# Response variable: response_variable (replace with actual trait name)
####################################################################

## M1: Homogeneous compound symmetry (CS) for genetic effects + IDV residual --
M1 <- asreml(
  fixed    = response_variable ~ Year + Rep + Year:Rep,
  random   = ~ Genotype + Genotype:Year + plot,
  data     = data,
  maxiter = 100,
  na.action = na.method(x = "include", y = "include")
)
M1 <- update(M1)

## M2: Diagonal genetic covariance + IDV residual ------------------------------
M2 <- asreml(
  fixed    = response_variable ~ Year + Year:Rep,
  random   = ~ Genotype:idh(Year) + plot,
  data     = data,
  maxiter = 100,
  na.action = na.method(x = "include", y = "include")
)
M2 <- update(M2)

## M3: Heterogeneous compound symmetry (CSH) + IDV residual --------------------
M3 <- asreml(
  fixed    = response_variable ~ Year + Rep + Year:Rep,
  random   = ~ Genotype:corh(Year) + plot,
  data     = data,
  maxiter = 100,
  na.action = na.method(x = "include", y = "include")
)
M3 <- update(M3)

## M4: Unstructured genetic covariance (UN) + IDV residual ---------------------
M4 <- asreml(
  fixed    = response_variable ~ Year + Rep + Year:Rep,
  random   = ~ Genotype:corgh(Year) + plot,
  data     = data,
```

```r
  maxiter = 100,
  na.action = na.method(x = "include", y = "include")
)
M4 <- update(M4)

## M5: Diagonal genetic covariance + heterogeneous residual (DIAG) -------------
M5 <- asreml(
  fixed     = response_variable ~ Year + Rep + Year:Rep,
  random    = ~ Genotype:idh(Year) + plot,
  residual  = ~ dsum(~ id(units) | Year),
  data      = data,
  maxiter   = 100,
  na.action = na.method(x = "include", y = "include")
)
M5 <- update(M5)

## M6: Heterogeneous compound symmetry (CSH) + heterogeneous residual ----------
M6 <- asreml(
  fixed     = response_variable ~ Year + Rep + Year:Rep,
  random    = ~ Genotype:corh(Year) + plot,
  residual  = ~ dsum(~ id(units) | Year),
  data      = data,
  maxiter   = 100,
  na.action = na.method(x = "include", y = "include")
)
M6 <- update(M6)

## M7: Unstructured genetic covariance (UN) + heterogeneous residual -----------
M7 <- asreml(
  fixed     = response_variable ~ Year + Rep + Year:Rep,
  random    = ~ Genotype:corgh(Year) + plot,
  residual  = ~ dsum(~ id(units) | Year),
  data      = data,
  maxiter   = 100,
  na.action = na.method(x = "include", y = "include")
)
M7 <- update(M7)

####################################################################
# Derived Genetic Parameters from Model 7 (UN + DIAG) -------------------------
####################################################################

## Extract general inputs -----------------------------------------------------
years <- levels(data$Year)
ny <- length(years)
nr <- nlevels(data$Rep)

## Extract variance components -------------------------------------------------
vc_M7 <- summary(M7)$varcomp

# Genetic variances per year
varG <- sapply(1:ny, function(i) {
```

```r
  vc_M7[paste0("Genotype:Year!Year_", i), "component"]
})
names(varG) <- years

# Residual variances per year
varE <- sapply(1:ny, function(i) {
  vc_M7[paste0("Year_", i, "!R"), "component"]
})
names(varE) <- years

# Plot (permanent environmental) variance
varPlot <- vc_M7["plot", "component"]

## Year-specific repeatability ------------------------------------------------
# Repeatability of genotype means within each year

Repeatability_year <- (varG + varPlot) /
  (varG + varPlot + varE / nr)

print(Repeatability_year)

## Genetic variance-covariance matrix (G matrix) ------------------------------

# Initialize matrix
Gvcov <- matrix(0, nrow = ny, ncol = ny,
                dimnames = list(years, years))

# Diagonal elements (genetic variances)
diag(Gvcov) <- varG

# Off-diagonal elements (genetic covariances)
for(i in 1:(ny - 1)){
  for(j in (i + 1):ny){
    cor_ij <- vc_M7[
      paste0("Genotype:Year!Year!", j, ":!Year!", i, ".cor"),
      "component"
    ]
    cov_ij <- cor_ij * sqrt(varG[i] * varG[j])
    Gvcov[i, j] <- cov_ij
    Gvcov[j, i] <- cov_ij
  }
}

print(Gvcov)

## Cumulative repeatability across years --------------------------------------

# Mean genetic variance
meanVarG <- mean(diag(Gvcov))

# Mean genetic covariance
meanCovG <- mean(Gvcov[upper.tri(Gvcov)])

# Mean residual variance
meanVarE <- mean(varE)
```

```r

# Mean phenotypic variance
sigma2f_mean <- meanVarG +
                (meanCovG / ny) +
                varPlot +
                (meanVarE / (ny * nr))

# Cumulative repeatability
Repeatability_cumulative <- meanVarG / sigma2f_mean

print(paste("Cumulative repeatability:",
            round(Repeatability_cumulative, 3)))

## Relative selection efficiency by number of years ----------------------------
# Efficiency relative to selection based on one year

Efficiency_years <- sapply(1:ny, function(k) {
  sqrt(k / (1 + (k - 1) * Repeatability_cumulative))
})

names(Efficiency_years) <- paste0("Year_", 1:ny)
print(Efficiency_years)

## BLUPs for Genotype x Year ---------------------------------------------------

blup_M7 <- predict(
  M7,
  classify = "Genotype:Year"
)$pvals

# Mean BLUP per genotype across years
BLUP_mean <- blup_M7 %>%
  group_by(Genotype) %>%
  summarise(
    BLUP_mean = mean(predicted.value),
    .groups = "drop"
  )

print(head(BLUP_mean))

## Yield persistence index -----------------------------------------------------
# Persistence defined as stability of performance relative to the
# yearly ideotype (maximum BLUP per year)

# Identify yearly ideotype
ideotype <- blup_M7 %>%
  group_by(Year) %>%
  summarise(
    max_BLUP = max(predicted.value),
    .groups = "drop"
  )

# Squared distance from ideotype
distance_sq <- blup_M7 %>%
  left_join(ideotype, by = "Year") %>%
```

```
  mutate(
    deviation = predicted.value - max_BLUP,
    deviation_sq = deviation^2
  ) %>%
  group_by(Genotype) %>%
  summarise(
    sum_deviation_sq = sum(deviation_sq),
    .groups = "drop"
  )

# Persistence index (inverse of summed squared deviation)
Persistence <- distance_sq %>%
  mutate(
    Persistence_raw = 1 / sum_deviation_sq,
    Persistence_index = Persistence_raw / sum(Persistence_raw)
  ) %>%
  select(Genotype, Persistence_index)

print(head(Persistence))

## Final selection table -------------------------------------------------------
# Combined information: mean performance + persistence

Selection_Table <- Persistence %>%
  left_join(BLUP_mean, by = "Genotype") %>%
  arrange(desc(BLUP_mean))

print(head(Selection_Table, 10))

####################################################################
# End of script
####################################################################
```

Notes on Code Execution

1. The script assumes a tab-delimited text file named `phenotype_data.txt` containing columns for `Genotype`, `Year`, `Rep`, and the trait of interest (e.g., `response_variable`).

2. The auxiliary function `fa_outs.R` must be sourced from the public repository or saved locally. This function is used for model convergence checking and updating.

3. Model fitting may require several minutes depending on dataset size and computational resources. The `maxiter` argument can be adjusted if convergence is not achieved.

4. Variance component extraction relies on consistent naming conventions in ASReml-R output. Users should verify column names in the `summary(model)$varcomp` output if using different ASReml-R versions.

5. All derived metrics (repeatability, efficiency, persistence) are generalizable and can be applied to any multi-year genotype evaluation trial with appropriate data structure.

References

References

Butler, D. G., Cullis, B. R., Gilmour, A. R., Gogel, B. G., and Thompson, R. (2023). *ASReml-R reference manual*. VSN International Ltd., Hemel Hempstead, UK, version 4.2 edition.

R Core Team (2025). *R: A Language and Environment for Statistical Computing*. R Foundation for Statistical Computing, Vienna, Austria.
