# Supplementary material for: Modeling temporal genetic variability using mixed models improves yield stability and selection efficiency in Coffea canephora
Source: Front Plant Sci. 2026 May 12;17:1840043. doi: 10.3389/fpls.2026.1840043 (PMC13201153; doi:10.3389/fpls.2026.1840043)
Supplement: Supplementary file 2 [file DataSheet2.docx]

**Supplementary Table S2**. Variance components and genetic parameters estimated under the seven models evaluated for grain yield in *Coffea canephora* across four crop seasons (2022–2025).

| **Model** | **Σg** | **Σe** | **Year** | $\boldsymbol{\sigma}_{\boldsymbol{g}}^{\boldsymbol{2}}$ | $\boldsymbol{\sigma}_{\boldsymbol{gy}}^{\boldsymbol{2}}$ | $\boldsymbol{\sigma}_{\boldsymbol{p}}^{\boldsymbol{2}}$ | $\boldsymbol{\sigma}_{\boldsymbol{e}}^{\boldsymbol{2}}$ | $\boldsymbol{\sigma}_{\boldsymbol{ph}}^{\boldsymbol{2}}$ | $\boldsymbol{h}^{\boldsymbol{2}}$ | $\boldsymbol{h}_{\boldsymbol{mg}}^{\boldsymbol{2}}$ | $\boldsymbol{h}_{\boldsymbol{Cullis}}^{\boldsymbol{2}}$ | $\boldsymbol{r}$ | $\boldsymbol{\rho}$ **(within-year)** | $\boldsymbol{\rho}$ **(cumulative)** |
| --- | --- | --- | --- | --- | --- | --- | --- | --- | --- | --- | --- | --- | --- | --- |
| M1 | CS | IDV | 2022 | 296.062 | 737.3285 | 9.3073 | 527.8395 | 833.2088 | 0.3553 | 0.6151 | 0.6151 | 0.7843 | 0.6344 | 0.8903 |
| M1 | CS | IDV | 2023 | 296.062 | 737.3285 | 9.3073 | 527.8395 | 833.2088 | 0.3553 | 0.6151 | 0.6151 | 0.7843 | 0.6344 | 0.8903 |
| M1 | CS | IDV | 2024 | 296.062 | 737.3285 | 9.3073 | 527.8395 | 833.2088 | 0.3553 | 0.6151 | 0.6151 | 0.7843 | 0.6344 | 0.8903 |
| M1 | CS | IDV | 2025 | 296.062 | 737.3285 | 9.3073 | 527.8395 | 833.2088 | 0.3553 | 0.6151 | 0.6151 | 0.7843 | 0.6344 | 0.8903 |
| M2 | DIAG | IDV | 2022 | 1360.6916 | - | 21.5125 | 518.2153 | 1900.4193 | 0.716 | 0.8751 | 0.8751 | 0.9355 | 0.8889 | 0.9404 |
| M2 | DIAG | IDV | 2023 | 896.7428 | - | 21.5125 | 518.2153 | 1436.4706 | 0.6243 | 0.822 | 0.822 | 0.9066 | 0.8417 | 0.9404 |
| M2 | DIAG | IDV | 2024 | 760.4162 | - | 21.5125 | 518.2153 | 1300.1439 | 0.5849 | 0.7965 | 0.7965 | 0.8925 | 0.8191 | 0.9404 |
| M2 | DIAG | IDV | 2025 | 1068.7072 | - | 21.5125 | 518.2153 | 1608.435 | 0.6644 | 0.8462 | 0.8462 | 0.9199 | 0.8632 | 0.9404 |
| M3 | CSH | IDV | 2022 | 1392.2128 | - | 10.0147 | 526.4227 | 1928.6502 | 0.7219 | 0.8824 | 0.8824 | 0.9394 | 0.8888 | 0.8888 |
| M3 | CSH | IDV | 2023 | 980.6207 | - | 10.0147 | 526.4227 | 1517.0581 | 0.6464 | 0.8409 | 0.8409 | 0.917 | 0.8495 | 0.8888 |
| M3 | CSH | IDV | 2024 | 724.2224 | - | 10.0147 | 526.4227 | 1260.6598 | 0.5745 | 0.7961 | 0.7961 | 0.8922 | 0.8071 | 0.8888 |
| M3 | CSH | IDV | 2025 | 1062.6469 | - | 10.0147 | 526.4227 | 1599.0843 | 0.6645 | 0.8514 | 0.8514 | 0.9227 | 0.8594 | 0.8888 |
| M4 | UN | IDV | 2022 | 1373.636 | - | 9.3076 | 527.8579 | 1910.8015 | 0.7189 | 0.8812 | 0.8812 | 0.9387 | 0.8871 | 0.8903 |
| M4 | UN | IDV | 2023 | 900.5319 | - | 9.3076 | 527.8579 | 1437.6974 | 0.6264 | 0.8294 | 0.8294 | 0.9107 | 0.8379 | 0.8903 |
| M4 | UN | IDV | 2024 | 776.1827 | - | 9.3076 | 527.8579 | 1313.3482 | 0.591 | 0.8073 | 0.8073 | 0.8985 | 0.817 | 0.8903 |
| M4 | UN | IDV | 2025 | 1083.3552 | - | 9.3076 | 527.8579 | 1620.5207 | 0.6685 | 0.854 | 0.854 | 0.9241 | 0.8613 | 0.8903 |
| M5 | DIAG | DIAG | 2022 | 1305.7017 | - | 22.557 | 680.5706 | 2008.8294 | 0.65 | 0.8396 | 0.8286 | 0.9103 | 0.8541 | 0.9396 |
| M5 | DIAG | DIAG | 2023 | 924.7634 | - | 22.557 | 432.8381 | 1380.1584 | 0.67 | 0.8472 | 0.8395 | 0.9163 | 0.8678 | 0.9396 |
| M5 | DIAG | DIAG | 2024 | 807.4833 | - | 22.557 | 373.9278 | 1203.9681 | 0.6707 | 0.8458 | 0.84 | 0.9165 | 0.8694 | 0.9396 |
| M5 | DIAG | DIAG | 2025 | 1046.8209 | - | 22.557 | 580.9838 | 1650.3617 | 0.6343 | 0.8288 | 0.8198 | 0.9055 | 0.8467 | 0.9396 |
| M6 | CSH | DIAG | 2022 | 1331.6721 | - | 11.7737 | 690.4259 | 2033.8717 | 0.6547 | 0.8463 | 0.8345 | 0.9135 | 0.8537 | 0.8869 |
| M6 | CSH | DIAG | 2023 | 1018.984 | - | 11.7737 | 439.7721 | 1470.5297 | 0.6929 | 0.8655 | 0.8538 | 0.924 | 0.8755 | 0.8869 |
| M6 | CSH | DIAG | 2024 | 765.3186 | - | 11.7737 | 381.2571 | 1158.3494 | 0.6607 | 0.8464 | 0.8375 | 0.9152 | 0.8594 | 0.8869 |
| M6 | CSH | DIAG | 2025 | 1040.1169 | - | 11.7737 | 589.553 | 1641.4435 | 0.6337 | 0.8332 | 0.8233 | 0.9073 | 0.8426 | 0.8869 |
| M7* | UN | DIAG | 2022 | 1319.0346 | - | 10.1041 | 690.7034 | 2019.842 | 0.653 | 0.8459 | 0.8474 | 0.9205 | 0.8524 | 0.8898 |
| M7* | UN | DIAG | 2023 | 931.5453 | - | 10.1041 | 433.9081 | 1375.5575 | 0.6772 | 0.8576 | 0.8446 | 0.919 | 0.8669 | 0.8898 |
| M7* | UN | DIAG | 2024 | 822.202 | - | 10.1041 | 388.9035 | 1221.2096 | 0.6733 | 0.8547 | 0.8673 | 0.9313 | 0.8652 | 0.8898 |
| M7* | UN | DIAG | 2025 | 1060.94 | - | 10.1041 | 594.1749 | 1665.2189 | 0.6371 | 0.836 | 0.8354 | 0.914 | 0.8439 | 0.8898 |

CS, compound symmetry; CSH, heterogeneous compound symmetry; DIAG, heterogeneous diagonal; UN, unstructured; IDV, homogeneous independent residuals. σ²g, genetic variance; σ²gy, genotype-by-year interaction variance (estimated only under M1); σ²p, permanent plot variance; σ²e, residual variance; σ²ph, phenotypic variance; h², plot-level heritability; h²mg, genotype-mean heritability; H²Cullis, Cullis heritability (Cullis et al., 2006); r, selective accuracy; ρ (within-year), within-year repeatability = (σ²g + σ²p) / (σ²g + σ²p + σ²e/nr); ρ (cumulative), cumulative repeatability across years accounting for genetic covariances between seasons. H²Cullis and r for models M1–M4 (homogeneous residuals) were approximated via h²mg, as the prediction error variance stored in these model objects reflects an internal parameterisation of the homogeneous residual structure that is not directly comparable to the genotypic scale. For models M5–M7 (heterogeneous residuals), H²Cullis and r were calculated from the prediction error variance extracted directly from the fitted model objects. * indicates the selected model.
